# Genome-Wide Association Study in Two Cohorts from a Multi-generational Mouse Advanced Intercross Line Highlights the Difficulty of Replication Due to Study-Specific Heterogeneity

**DOI:** 10.1534/g3.119.400763

**Published:** 2020-01-24

**Authors:** Xinzhu Zhou, Celine L. St. Pierre, Natalia M. Gonzales, Jennifer Zou, Riyan Cheng, Apurva S. Chitre, Greta Sokoloff, Abraham A. Palmer

**Affiliations:** * Biomedical Sciences Graduate Program, University of California San Diego, La Jolla, CA, 92092,; † Department of Genetics, Washington University School of Medicine, St. Louis, MO, 63110,; ‡ Department of Human Genetics, University of Chicago, IL, 60637,; § Department of Computer Science, University of California, Los Angeles, CA, 90095,; ** Department of Psychiatry,; ‡‡ Institute for Genomic Medicine, University of California San Diego, La Jolla, CA, 92037 and; †† Department of Psychological & Brain Sciences, University of Iowa, Iowa City, IO, 52242

**Keywords:** GWAS, Mouse Genetics, Replication, Multiparent Advanced Generation Inter-Cross (MAGIC), multiparental, populations, MPP

## Abstract

There has been extensive discussion of the “Replication Crisis” in many fields, including genome-wide association studies (**GWAS**). We explored replication in a mouse model using an advanced intercross line (**AIL**), which is a multigenerational intercross between two inbred strains. We re-genotyped a previously published cohort of LG/J x SM/J AIL mice (F_34_; n = 428) using a denser marker set and genotyped a new cohort of AIL mice (F_39-43_; n = 600) for the first time. We identified 36 novel genome-wide significant loci in the F_34_ and 25 novel loci in the F_39-43_ cohort. The subset of traits that were measured in both cohorts (locomotor activity, body weight, and coat color) showed high genetic correlations, although the SNP heritabilities were slightly lower in the F_39-43_ cohort. For this subset of traits, we attempted to replicate loci identified in either F_34_ or F_39-43_ in the other cohort. Coat color was robustly replicated; locomotor activity and body weight were only partially replicated, which was inconsistent with our power simulations. We used a random effects model to show that the partial replications could not be explained by Winner’s Curse but could be explained by study-specific heterogeneity. Despite this heterogeneity, we performed a mega-analysis by combining F_34_ and F_39-43_ cohorts (n = 1,028), which identified four novel loci associated with locomotor activity and body weight. These results illustrate that even with the high degree of genetic and environmental control possible in our experimental system, replication was hindered by study-specific heterogeneity, which has broad implications for ongoing concerns about reproducibility.

Genome-wide association studies (**GWAS**) in model organisms can use genetically identical cohorts phenotyped under extremely similar conditions, which would be expected to enhance the success of replication. We sought to investigate replication in model organism GWAS using a mouse advanced intercross line (**AIL**). The use of GWAS in model organisms such as mice (Talbot *et al.* 1999; Demarest *et al.* 2001; Yalcin *et al.* 2004; Valdar *et al.* 2006; Ghazalpour *et al.* 2008; Samocha *et al.* 2010; Churchill *et al.* 2012; Collaborative Cross Consortium 2012; Parker *et al.* 2012, 2016; Svenson *et al.* 2012; Carbonetto *et al.* 2014; Chesler 2014; Coyner *et al.* 2014; Gatti *et al.* 2014; Nicod *et al.* 2016; Hernandez Cordero *et al.* 2018, 2019), rats (Baud *et al.* 2014), chickens (Besnier *et al.* 2011; Johnsson *et al.* 2018), fruit flies (King *et al.* 2012; Mackay *et al.* 2012; Kislukhin *et al.* 2013; Marriage *et al.* 2014; Vonesch *et al.* 2016), *C. elegans* (Doitsidou *et al.* 2016) and various plant species (Rishmawi *et al.* 2017; Cockram and Mackay 2018; Diouf *et al.* 2018) has become increasingly common over the last decade. These mapping populations can further be categorized as multi-parental crosses, which are created by interbreeding two or more inbred strains, and various outbred populations, in which the founders are of unknown provenance. An F_2_ cross between two inbred strains is the prototypical mapping population; however, F_2_s provide poor mapping resolution (Parker and Palmer 2011). To improve mapping resolution, Darvasi and Soller (Darvasi and Soller 1995) proposed the creation of advanced intercross lines (**AILs**), which are produced by intercrossing F_2_ mice for additional generations. AILs accumulate additional crossovers with every successive generation, leading to a population with shorter linkage disequilibrium (**LD**) blocks, which improves mapping precision, albeit at the expense of power (Parker and Palmer 2011; Gonzales and Palmer 2014).

The longest running mouse AIL was generated by crossing LG/J and SM/J inbred strains, which had been previously selected for large and small body size prior to inbreeding and subsequent intercrossing. We obtained this AIL in 2006 at generation 33 from Dr. James Cheverud (Jmc: LG,SM-G_33_). Since then, we have collected genotype and phenotype information from multiple generations, including F_34_ (Cheng *et al.* 2010; Lionikas *et al.* 2010; Samocha *et al.* 2010; Parker *et al.* 2011, 2014; Bartnikas *et al.* 2012; Carroll *et al.* 2017; Gonzales *et al.* 2018) and F_39_-F_43_. Our previous publications using the F_34_ generation employed a custom Illumina Infinium genotyping microarray to obtain genotypes for 4,593 SNPs (Cheng *et al.* 2010; Parker *et al.* 2014); we refer to this set of SNPs as the ‘sparse markers’. Those genotypes were used to identify significant associations for numerous traits, including locomotor activity in response to methamphetamine (Cheng *et al.* 2010), pre-pulse inhibition (Samocha *et al.* 2010), muscle weight (Lionikas *et al.* 2010; Hernandez Cordero *et al.* 2019), body weight (Parker *et al.* 2011), open field (Parker *et al.* 2014), conditioned fear (Parker *et al.* 2014), red blood cell parameters (Bartnikas *et al.* 2012), and muscle weights (Carroll *et al.* 2017). Although not previously published, we also collected phenotype information from the F_39-43_ generations, including body weight, fear conditioning, locomotor activity in response to methamphetamine, and the light dark test for anxiety.

While the prior GWAS using the F_34_ generation detected many significant loci, the sparsity of the markers likely precluded the discovery of some true loci and also made it difficult to clearly define the boundaries of the loci that we did identify. For example, Parker *et al.* conducted an integrated analysis of F_2_ and F_34_ AILs (Parker *et al.* 2011). One of their body weight loci spanned from 87.93–102.70 Mb on chromosome 14. Denser markers might have more clearly defined the implicated region.

In the present study, we used genotyping-by-sequencing (**GBS**), which is a reduced-representation sequencing method (Davey *et al.* 2011; Elshire *et al.* 2011; Fitzpatrick *et al.* 2013), to obtain a much denser set of SNPs in the F_34_ cohort and, for the first time, genotyped mice from the F_39-43_ generations. With this denser set of SNPs, we attempted to identify novel loci in the F_34_ cohort that were not detected using the sparse SNPs. We also performed GWAS using the mice from the F_39-43_ AILs. We explored whether imputation from the array SNPs could have provided the additional coverage we obtained using the denser GBS genotypes. Because F_39-43_ AILs are the direct descendants of the F_34_, they are uniquely suited to serve as a replication population for GWAS in the F_34_ generation. For the subset of traits measured in both cohorts, we attempted to replicate the results discovered in one cohort in the other. To set our expectations for replication, we performed simulations to estimate the power for these replication studies. Because the actual rate of replication was lower than predicted by the power analysis, we used a random effects model to evaluate the role of Winner’s Curse and study-specific heterogeneity in the low rate of replication. Finally, we also performed a mega-analysis on a subset of traits common to both cohorts.

## Materials and Methods

### Animals

All mice used in this study were members of the LG/J x SM/J AIL that was originally created by Dr. James Cheverud (Loyola University Chicago, Chicago, IL). This AIL has been maintained in the Palmer laboratory since generation F_33_. Age and exact number of animals tested in each phenotype are described in Table S1. Several previous publications (Samocha *et al.* 2010; Cheng *et al.* 2010; Parker *et al.* 2014; Lionikas *et al.* 2010; Carroll *et al.* 2017; Parker *et al.* 2011; Bartnikas *et al.* 2012) have reported association analyses of the F_34_ mice (n = 428). No prior publications have described the F_39-43_ generations (n = 600). The sample size of F_34_ mice reported in this study (n = 428) is smaller than that in previous publications of F_34_ (n = 688) because we only genotyped a subset of F_34_ animals using GBS.

### F_34_, F_39-43_ Phenotypes

All phenotypes are listed in Table S1. We have previously described the phenotyping of F_34_ animals for locomotor activity and locomotor response to methamphetamine (Cheng *et al.* 2010), fear conditioning (Parker *et al.* 2014), open field (Parker *et al.* 2014), coat color, body weight (Parker *et al.* 2011), complete blood counts (Bartnikas *et al.* 2012), heart and tibia measurements (Lionikas *et al.* 2010), muscle weight (Lionikas *et al.* 2010). Iron content in liver and spleen, which have not been previously reported in these mice, was measured by atomic absorption spectrophotometry, as described in Gardenghi *et al.* (Gardenghi *et al.* 2007) and Graziano, Grady and Cerami (Graziano *et al.* 1974). Although the phenotyping of F_39-43_ animals has not been previously reported, we followed previously published protocols for locomotor activity and locomotor response to methamphetamine (Cheng *et al.* 2010), coat color, body weight (Parker *et al.* 2011), and light/dark test for anxiety (Sittig *et al.* 2016). We point out here that even though “locomotor activity” was measured in both the F_34_ and F_39-43_ using the Versamax software (AccuScan Instruments, Columbus, OH), “open field” in the F_34_ cohort was also measured using Versamax, whereas “open field” in the F_39-43_ cohort was measured using the EthoVision XT software (Noldus system; (Noldus *et al.* 2001)). Because there are meaningful differences in these experimental procedures, we did not attempt to use “open field” data for replication. In summary, we performed GWAS on all traits collected in individual cohorts. For the replication analysis between the F_34_ and F_39-43_ cohorts, we only directly compared a number of traits that had been measured in both cohorts: body weight, two Mendelian coat color traits (albino and agouti), and three locomotor activity traits (locomotor activity on day 1 and on day 2, and activity on day 3 following a methamphetamine injection).

### F_34_ AIL Array Genotypes

F_34_ animals had been genotyped on a custom SNP array on the Illumina Infinium platform (Cheng *et al.* 2010; Parker *et al.* 2014), which yielded a set of 4,593 SNPs on autosomes and X chromosome that we refer to as ‘sparse SNPs’.

### F_34_ and F_39-43_ GBS Genotypes

F_34_ and F_39-43_ animals were genotyped using genotyping-by-sequencing (**GBS**), which is a reduced-representation genome sequencing method (Parker *et al.* 2016; Gonzales *et al.* 2017). We used the same protocol for GBS library preparation that was described in Gonzales *et al.* (Gonzales *et al.* 2017). We called GBS genotype probabilities using ANGSD (Korneliussen *et al.* 2014). GBS identified 1,667,920 autosomal and 43,015 X-chromosome SNPs. To fill in missing genotypes at SNPs where some but not all mice had calls, we ran within-sample imputation using Beagle v4.1, which generated hard call genotypes as well as genotype probabilities (Browning and Browning 2007). After imputation, only SNPs that had dosage *r^2^* > 0.9 were retained. We removed SNPs with minor allele frequency < 0.1 and SNPs with *P* < 1.0×10^−6^ in the Chi-square test of Hardy–Weinberg Equilibrium (**HWE**) (Table S2). All phenotype and GBS genotype data are deposited in GeneNetwork2 (http://gn2.genenetwork.org/).

### QC of individuals

We have found that large genetic studies are often hampered by cross-contamination between samples and sample mix-ups. We used four features of the data to identify problematic samples: heterozygosity distribution, proportion of reads aligned to sex chromosomes, pedigree/kinship, and coat color. We first examined heterozygosity across autosomes and removed animals where the proportion of heterozygosity was more than 3 standard deviations from the mean (Figure S1). Next, we sought to identify animals in which the recorded sex did not agree with the sequencing data. We compared the ratio of reads mapped to the X and Y chromosomes. The 95% CI for this ratio was 196.84 to 214.3 in females and 2.13 to 2.18 in males. Twenty-two F_34_ and F_39-43_ animals were removed because their sex (as determined by reads ratio) did not agree with their recorded sex; we assumed this discrepancy was due to sample mix-ups. To further identify mislabeled samples, we calculated kinship coefficients based on the full AIL pedigree using QTLRel. We then calculated a genetic relatedness matrix (**GRM**) using IBDLD (Abney 2008; L. Han and Abney 2011), which estimates identity by descent using genotype data. The comparison between pedigree kinship relatedness and genetic kinship relatedness identified seven pairs of animals that showed obvious disagreement between kinship coefficients and the GRM, these animals were excluded from further analysis. Lastly, we excluded 14 F_39-43_ animals that showed discordance between their recorded coat color and their genotypes at markers flanking *Tyr*, which causes albinism in mice. The numbers of animals filtered at each step are listed in Table S2. Some animals were detected by more than one QC step, substantiating our evidence that these samples were erroneous.

At the end of SNP and sample filtering, we had 59,561 autosomal and 831 X chromosome SNPs in F_34_, 58,966 autosomal and 824 X chromosome SNPs in F_39-43_, and 57,635 autosomal and 826 X chromosome SNPs in the combined F_34_ and F_39-43_ set (Table S2). GBS genotype quality was estimated by examining concordance between the 66 SNPs that were present in both the array and GBS genotyping results (Figure S3).

### LD decay

Average LD (*r^2^*) was calculated using allele frequency matched SNPs (MAF difference < 0.05) within 100,000 bp distance, as described in Parker *et al.* (Parker *et al.* 2016).

### Imputation to LG/J and SM/J reference panels

F_34_ array genotypes (n = 428) and F_34_ GBS genotypes (n = 428) were imputed to LG/J and SM/J whole genome sequence data (Nikolskiy *et al.* 2015) using BEAGLE (Browning and Browning 2007). For F_34_ array imputation, we used a large window size (100,000 SNPs and 45,000 SNPs overlap). Imputation to reference panels yielded 4.3 million SNPs for F_34_ array and F_34_ GBS imputed sets. Imputed SNPs with r^2^ > 0.9, MAF > 0.1, HWE p-value > 1.0×10^−6^ were retained, resulting in 4.1M imputed F_34_ GBS SNPs and 4.3M imputed F_34_ array SNPs.

### Genome-wide association analysis (GWAS)

We used the linear mixed model, as implemented in GEMMA (Zhou and Stephens 2012), to perform a GWAS that accounted for the complex familial relationships among the AIL mice (Cheng *et al.* 2010; Gonzales *et al.* 2017). We used the leave-one-chromosome-out (**LOCO**) approach to calculate the GRM, which effectively circumvented the problem of proximal contamination (Cheng *et al.* 2013). We used the univariate linear mixed model described in Zhou and Stephens (Zhou and Stephens 2012):

$$y=W\alpha +x\beta +u+\varepsilon ;u∼MVN_{n}\left(0,\lambda \tau^{-1}K\right),\varepsilon ∼MVN_{n}\left(0,\tau^{-1}I_{n}\right),$$

where y is a n-vector of traits for n individuals; W is a n×c matrix of covariates (fixed effects); α is a c-vector of the corresponding coefficients; x is an n-vector of genotypes; β is the effect size of the genotype; u is an n-vector of random effects; ε is an n-vector of errors; $\tau^{-1}$is the variance of the residual errors; λ is the ratio between the two variance components; K is a known n × n relatedness matrix and $I_{n}$ is an n × n identity matrix. $MVN_{n}$ stands for the n-dimensional multivariate normal distribution (Zhou and Stephens 2012).

Separate GWAS were performed using the F_34_ array genotypes, the F_34_ GBS genotypes, and the F_39-43_ GBS genotypes. Apart from coat color (binary trait), raw phenotypes were quantile normalized prior to analysis. Coat color traits were coded as follows: albino: 1 = white, 0 = non-white; agouti: 1 = tan, 0 = black, NA = white. Because F_34_ AIL had already been studied, we used the same covariates as described in Cheng *et al.* (Cheng *et al.* 2010) in order to examine whether our array and GBS GWAS would replicate their findings. We included sex and body weight as covariates for locomotor activity traits (see covariates used in (Cheng *et al.* 2010)) and sex, age, and coat color as covariates for fear conditioning and open field test in F_34_ AILs (see covariates used in (Parker *et al.* 2014)). We used sex and age as covariates for all other phenotypes. Covariates for each analysis are shown in Table S1. Finally, we performed mega-analysis of F_34_ and F_39-43_ animals (n = 1,028) for body weight, coat color, and locomotor activity, since these traits were measured in the same way in both cohorts. We quantile transformed all continuous phenotypes in each cohort and then combined the transformed phenotypes for the mega-analysis (coat color traits were not quantile normalized because they are binary).

### Identifying dubious SNPs

Some significant SNPs in the F_34_ GWAS were dubious because the flanking SNPs, which would have been expected to be in high LD with the significant SNP (a very strong assumption in an AIL), did not have high -log10(p) values. We only examined SNPs that obtained significant p-values; close examinations revealed that these SNPs had dubious ratios of heterozygotes to homozygotes calls and had corresponding HWE p-values that were close to our 1.0×10^−6^ threshold (Table S3). We chose the 1.0×10^−6^ as the filter threshold of the HWE p-values based on a gene-dropping exercise. We used the F_33-34_ family pedigree and the F_34_ genetic map to simulate the genotypes in F_34_ (QTLRel; (Cheng *et al.* 2011)). The p-value of the chi-square test for Hardy-Weinberg equilibrium in the simulated F_34_ population was 7.24329×10^−06^, which was close to the HWE threshold used in Gonzales *et al.* (Gonzales *et al.* 2018). To avoid counting these as novel loci, we removed those SNPs prior to summarizing our results as they likely reflected genotyping errors.

### Selecting independent significant SNPs

To identify independent “lead loci” among significant GWAS SNPs that surpassed the significance threshold, we used the LD-based clumping method in PLINK v1.9. We empirically chose clumping parameters (*r^2^* = 0.1 and sliding window size = 12,150kb) that gave us a conservative set of independent SNPs (Table S4). For the coat color phenotypes, we found that multiple SNPs remained significant even after LD-based clumping, presumably due to the extremely significant associations at these Mendelian loci. In these cases, we used a stepwise model selection procedure in GCTA (Yang *et al.* 2011) and performed association analyses conditioning on the most significant SNPs.

### Significance thresholds

We used MultiTrans to set significance thresholds for GWAS (B. Han *et al.* 2009; Joo *et al.* 2016). MultiTrans is a method that assumes multivariate normal distribution of the phenotypes, which in LMM models, contain a covariance structure due to various degrees of relatedness among individuals. We were curious to see whether MultiTrans produced significance thresholds that were different from the thresholds we obtained from a standard permutation test (‘naïve permutation’ as per Cheng *et al.* (Cheng *et al.* 2013)). We performed 1,000 permutations using the F_34_ GBS genotypes and the phenotypic data from locomotor activity (days 1, 2, and 3). We found that the 95^th^ percentile values for these permutations were 4.65, 4.79, and 4.85, respectively, which were very similar to 4.85, the threshold obtained from MultiTrans using the same data. Thus, the thresholds presented here were obtained from MultiTrans but are similar (if anything slightly more conservative) to the thresholds we would have obtained had we used permutation. Because the effective number of tests depends on the number of SNPs and the specific animals used in GWAS, we obtained a unique adjusted significance threshold for each SNP set in each animal cohort (Table S5).

### Credible set analysis

We followed the method described in (Wellcome Trust Case Control Consortium *et al.* 2012). Credible set analysis is a Bayesian method of selecting an interval of SNPs that are likely to contain the causal SNPs; we used LD r^2^ threshold = 0.8, posterior probability =0.99. The R script could be found on GitHub: https://github.com/hailianghuang/FM-summary/blob/master/getCredible.r

### Power analysis

To estimate the power of replication of a SNP from the discovery set in the replication set, we simulated GWAS with 50 varying effect sizes for the discovery SNP using the LMM model. We first fit the trait in a null model (*i.e.*, no genotype effect), and obtained estimates of model parameters including the intercept and the genetic variance component. Using these model parameters, we added the genotype effect to the random numbers generated from the null model to recreate a trait. For each simulated effect size, we scanned every simulated trait 2,500 times and examined the ratio of association tests whose test statistics surpassed the significance thresholds (both the genome-wide significance threshold for the cohort and the nominal p-value of 0.05).

### Replication analysis between F_34_ and F_39-43_ GWAS studies

We modeled the replication between F_34_ and F_39-43_ GWAS studies using two random effects models (Zou *et al.* 2019). Both models take as input a set of z-scores for variants computed from an association study (“summary statistics”).

The **WC** model accounts only for Winner’s Curse. We assume that there is a shared genetic effect (λ) that is responsible for the observed association signal in both studies. To model random noise contributing to Winner’s Curse, we model the summary statistics for each variant k from the discovery and replication studies as normally distributed random variables ($s_{k}^{\left(1\right)}∼\;N\left(\lambda ,\;\;1\right)$ and $s_{k}^{\left(2\right)}∼\;N\left(\lambda ,\;\;1\right)$, respectively). We define the prior probability of the true genetic effect to be $\lambda ∼N\left(0,\;\sigma_{g}^{2}\right)$, where the variance in the true genetic effect is learned through a maximum likelihood procedure. We correct for the effect of Winner’s Curse in the discovery study by computing the conditional distribution of the replication summary statistic given the discovery summary statistic.

The **WC+C** model accounts for Winner’s Curse and study-specific heterogeneity. In this model, we partition the total effect sizes observed into genetic effects (λ) and study-specific effects ($\delta^{\left(1\right)}$ and $\delta^{\left(2\right)}$). We model the statistics for each variant k from the initial and discovery studies as normally distributed random variables ($s_{k}^{\left(1\right)}∼\;N\left(\lambda +\;\delta^{\left(1\right)},\;\;1\right)$ and $s_{k}^{\left(2\right)}∼\;N\left(\lambda \;+\;\delta^{\left(2\right)},\;\;1\right)$, respectively). In addition to the prior on the genetic effect defined in the **WC** model, we define the prior probabilities of the study-specific effects to be $\delta^{\left(1\right)}∼N\left(0,\;\sigma_{c_{1}}^{2}\right)$, and $\delta^{\left(2\right)}∼N\left(0,\;\sigma_{c_{2}}^{2}\right)$, where the variance parameters are learned through a maximum likelihood procedure. We correct for the effect of Winner’s Curse in the discovery study and study-specific effects by computing the conditional distribution of the replication summary statistic given the discovery summary statistic.

We applied each of these models once using F_34_ as the discovery study and once using F_39-43_ as the discovery study. We used the genome-wide significance thresholds in Table S5 to identify variants in each discovery study and used the results as input to the random effects models. We then used a Bonferroni corrected threshold (*P* = 0.05/M) for the replication study, where M is the number of genome-wide significant variants in the initial study. We computed the “empirical replication rate” as the proportion of variants passing the genome-wide significant threshold in the discovery study that also passed this Bonferroni corrected threshold in the replication study. Since the estimation of the model parameters requires at least two variants, we only applied this method to phenotypes with at least two genome-wide significant variants in the discovery study.

To assess how well the **WC** and **WC+C** models explained the observed patterns of replication, we computed the predicted replication rates under each model. For each variant that passed the genome-wide significant threshold in the discovery study, we used the conditional distributions previously learned to compute the probability that the variant passed the Bonferroni corrected threshold in the replication study. For each phenotype, we computed the average of these predicted replication rates and compared this average to the empirical replication rates.

### Genetic correlation and heritability estimates between F_34_ and F_39-43_ phenotypes

Locomotor activity, body weight, and coat color traits had been measured in both F_34_ and F_39-43_ populations. We calculated both SNP heritability and genetic correlations between F_34_ and F_39-43_ animals using GCTA-GREML analysis and GCTA bivariate GREML analysis (Yang *et al.* 2011).

### LocusZoom Plots

LocusZoom plots were generated using the standalone implementation of LocusZoom (Pruim *et al.* 2010), using LD scores calculated from PLINK v.1.9–ld option and mm10 gene annotation file downloaded from UCSC Genome Browser.

### Data availability

All relevant data are within the paper and its Supporting Information files. Genotypes and phenotypes of F_34_ (“LGSM AI G34 Palmer (Array)”: GN655; “LGSM AI G34 Palmer (GBS)”: GN656), F_39-43_ (“LGSM AI G39-43 Palmer (GBS)”: GN657), and mega-analysis cohort (“LGSM AI G34 G39-43 Palmer (GBS)”: GN654) of AIL are uploaded to GeneNetwork2 (http://gn2.genenetwork.org/). Code used to perform the analyses is included in the supplementary materials on figshare: https://doi.org/10.25387/g3.11674221.

## Results

**Figure 1:**
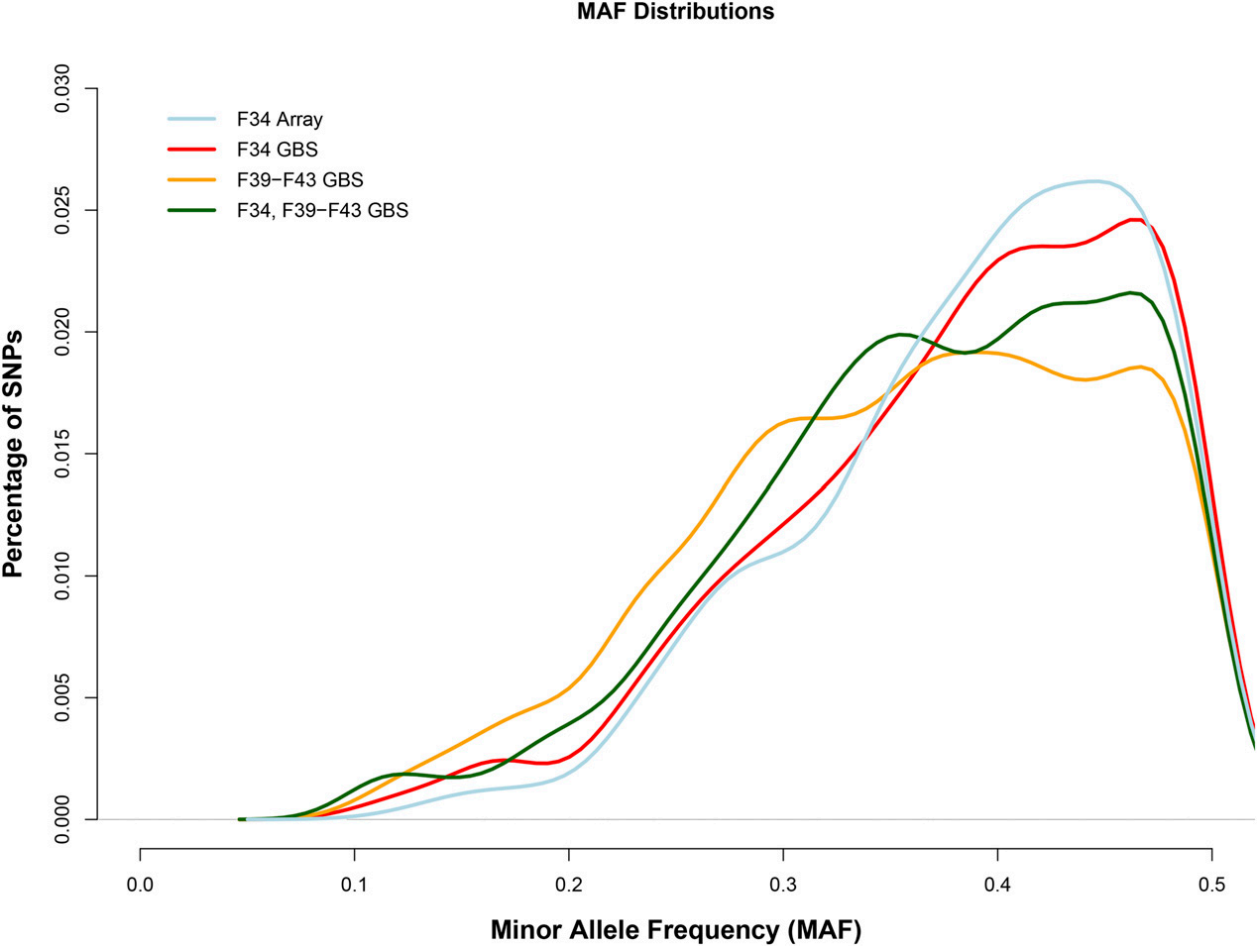
Minor allele frequency (MAF) distributions for F_34_ array, F_34_ GBS, F_39_-F_43_ GBS, and F_34_ and F_39_-F_43_ GBS SNP sets. The average MAF of those 4,593 array SNPs was 0.388; the average MAF of the 60,392 GBS-derived SNPs in 428 F_34_ AIL mice was 0.382; the average MAF of the 59,790 GBS-derived SNPs in 600 F_39-43_ AIL mice was 0.358; the average MAF of the 58,461 GBS-derived SNPs that existed in both F_34_ and F_39-43_ AIL mice was 0.370 (Table S2). MAF distributions are highly comparable between AIL generations.

We used 214 males and 214 females from generation F_34_ (Aap:LG,SM-G34) and 305 males and 295 females from generations F_39-43_. For the F_34_ AIL 79 traits were available from previous published and unpublished work; for the F_39-43_ AIL 49 unpublished traits were available (Table S1). F_34_ mice had been previously genotyped on a custom SNP array (Cheng *et al.* 2010; Parker *et al.* 2014). The average minor allele frequency (**MAF**) of those 4,593 array SNPs was 0.388 (Figure 1). To obtain a denser set of SNP markers, we used GBS in F_34_ and F_39-43_ AIL mice. Since data on the F_39-43_ AIL mice had been collected over the span of approximately two years, we carefully considered the possibility of sample contamination and sample mislabeling (Toker *et al.* 2016) and removed these samples (see Methods; Figure S1 and S2). The final SNP sets included 60,392 GBS-derived SNPs in 428 F_34_ AIL mice, 59,790 GBS-derived SNPs in 600 F_39-43_ AIL mice, and 58,461 GBS-derived SNPs that existed in both F_34_ and F_39-43_ AIL mice (Table S2). The MAF for the GBS SNPs was 0.382 in F_34_, 0.358 in F_39-43_, and 0.370 in F_34_ and F_39-43_ (Figure 1). There were 66 SNPs called from our GBS data that were also present on the genotyping array. The genotype concordance rate for those 66 SNPs, which reflects the sum of errors from both sets of genotypes, was 95.4% (Figure S3). We found that LD decay rates using F_34_ array, F_34_ GBS, F_39-43_ GBS, and F_34_ and F_39-43_ GBS genotypes were generally similar to one another, though levels of LD using the GBS genotypes appear to be slightly reduced in the later generations of AILs (Figure S4).

### GBS genotypes produced more significant associations than array genotypes in F_34_

We used a linear mixed model (**LMM**) as implemented in GEMMA (Zhou and Stephens 2012) to perform GWAS. We used the leave-one-chromosome-out (**LOCO**) approach to address the problem of proximal contamination, as previously described (Listgarten *et al.* 2012; Cheng *et al.* 2013; Yang *et al.* 2014; Gonzales *et al.* 2017). We performed GWAS using both the sparse array SNPs and the dense GBS SNPs to determine whether additional SNPs would produce more genome-wide significant associations. Autosomal and X chromosome SNPs were included in all GWAS. We obtained a significance threshold for each SNP set using MultiTrans (B. Han *et al.* 2009; Joo *et al.* 2016).To select independently associated loci (“lead loci”), we used an LD-based clumping method implemented in PLINK to group SNPs that passed the adjusted genome-wide significance thresholds over a large genomic region flanking the index SNP (Purcell *et al.* 2007). Applying the most stringent clumping parameters (*r^2^* = 0.1 and sliding window size = 12,150kb, Table S4), we identified 109 significant lead loci in 49 out of 79 F_34_ phenotypes using the GBS SNPs (Table S7). In contrast, we identified 83 significant lead loci in 45 out of 79 F_34_ phenotypes using the sparse array SNPs (Table S6, Table S7). Among the loci identified in the F_34_, 36 were uniquely identified using the GBS genotypes, whereas 11 were uniquely identified using the array genotypes. These unique loci could be explained by the disparity of the marker density between the GBS and array genotypes. Some unique loci captured haplotype blocks that were not picked up in the other SNP set. Other unique loci were only slightly above the significance threshold in one SNP set where the corresponding loci in the other SNP set had sub-threshold significance (*i.e.*, p-value ∼10^−5^ but below the significance threshold of the cohort; Table S7). Overall, GBS SNPs consistently yielded more significant lead loci compared to array SNPs regardless of the clumping parameter values (Table S4), indicating that a dense marker panel was able to detect more association signals compared to a sparse marker panel.

**Figure 2:**
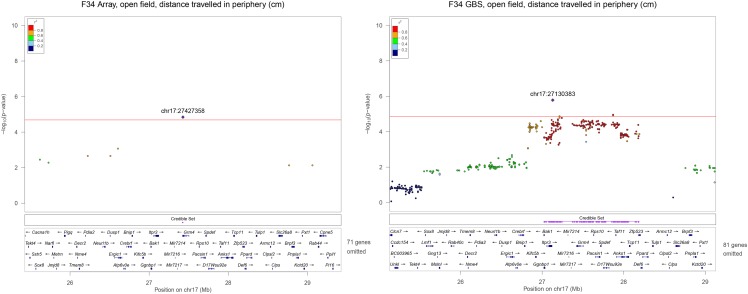
Significant loci on chromosome 17 for open field, distance traveled in periphery in F_34_ AIL. As exemplified in this pair of LocusZoom plots, GBS SNPs defined the boundaries of the loci much more precisely than array SNPs. GBS SNPs that are in high LD (r^2^ > 0.8, red dots) with lead SNP chr17:27130383 resides between 27 ∼28.3 Mb. In contrast, too few SNPs are present in the array plot to draw any definitive conclusion about the boundaries or LD pattern in this region. Purple track shows the credible set interval. LocusZoom plots for all loci identified in this paper are in Figure S8.

To determine the boundaries of each locus, we performed a Bayesian-framework credible set analysis, which estimated a posterior probability for association at each SNP (*r^2^* threshold = 0.8, posterior probability threshold = 0.99; (Wellcome Trust Case Control Consortium *et al.* 2012)). The physical positions of the SNPs in the credible set were used to determine the boundaries of each locus. As expected, the greater density of the GBS genotypes allowed us to better define each interval. For instance, the lead locus at chr17:27130383 was associated with distance traveled in periphery in the open field test in F_34_ AILs (Figure 2). However, no SNPs were genotyped between 26.7 and 28.7 Mb in the array SNPs, which makes the size of this LD block ambiguous. In contrast, the LocusZoom plot portraying GBS SNPs in the same region shows that SNPs in high LD with chr17:27130383 are between 27 Mb and 28.3 Mb. The more accurate definition of the implicated intervals allowed us to better refine the list of the coding genes and non-coding variants associated with the phenotype (Table S6).

In our prior studies using the sparse marker set, we did not attempt to increase the number of available markers by using imputation. Therefore, we examined whether the disparity between the numbers of loci identified by the two SNP sets could be resolved by imputation, which should increase the number of markers available for GWAS. We used LG/J and SM/J whole genome sequencing data as reference panels (Nikolskiy *et al.* 2015) and performed imputation on array and GBS SNPs using Beagle v4.1 (Browning and Browning 2007). After QC filtering, we obtained 4.3M SNPs imputed from the array SNPs and 4.1M SNPs imputed from the GBS SNPs. More imputed GBS SNPs were filtered out because GBS SNPs were called from genotype probabilities, thus introducing uncertainty in imputed SNPs. We found that imputed array genotypes and imputed GBS genotypes did not meaningfully increase the number of loci discovered, presumably because the utility of imputation is inherently limited in a two-strain cross.

Under a polygenic model where a large number of additive common variants contribute to a complex trait, heritability estimates could be higher when more SNPs are considered (Yang *et al.* 2017). Given that there were more GBS SNPs than array SNPs, we used autosomal SNPs to examine whether GBS SNPs would generate higher SNP heritability estimates compared to the sparse array SNPs. Heritability estimates were similar for the two SNP sets, with the exception of agouti coat color, which showed marginally greater heritability for the GBS SNPs (Figure S5; Table S8). Our results show that while the denser GBS SNP set was able to identify more genome-wide significant loci, greater SNP density did not improve the polygenic signal.

### Partial replication of loci identified in F_34_ or F_39-43_ and mega-analysis

We identified 25 genome-wide significant loci for 21 phenotypes in the F_39-43_ cohort (Table S9). A subset of those traits: coat color, body weight, and locomotor activity, were also phenotyped in the F_34_ AILs. To assess replication, we determined whether the loci that were significant in one cohort (either F_34_ or F_39-43_) would also be significant in the other. We termed the cohort in which a locus was initially discovered as its “discovery set” and the cohort we attempted replication in as the “replication set” (Table 1). Coat color phenotypes (both albino and agouti) are Mendelian traits and thus served as positive controls. All coat color and body weight loci were replicated. The three body weight loci identified in the F_34_ were replicated at nominal levels of significance (*P* < 0.05) in F_39-43_; similarly, one body weight locus identified in F_39-43_ was replicated in F_34_ (*P* < 0.05). However, none of the locomotor activity loci were replicated in the reciprocal (replication) cohorts.

**Table 1:** Replication of significant SNPs between F_34_ and F_39-43_ AIL association analyses. “Discovery set” indicates the AIL generation that significant SNPs were identified. “Replication set” shows the association p-value, β estimates, etc. of the “discovery set” significant SNPs in the replication AIL generation. SNPs that replicated (**p** < 0.05, same sign for the beta) between F_34_ and F_39-43_ are in bold italics, SNPs that replicated at the genome-wide threshold (see Table S5) are bold, italic and underlined. Genetic correlations (rG) for phenotypes measured in both F_34_ and F_39-43_ are listed (see also Table S11)

			Discovery set					Replication set				
Phenotype	rG(s.e.)	SNP	p	-log10(p)	af	beta	se	p	-log10(p)	af	beta	se
			F_34_ GBS					F_3943_ GBS replicate				
Body weight	0.711(0.25)*	***chr4.66414508***	***8.58×10^−8^***	***7.07***	0.419	−0.25	0.05	***3.55×10^−3^***	***2.45***	0.406	−0.13	0.04
		***chr6.81405109***	***6.22×10^−6^***	***5.21***	0.497	0.21	0.05	***3.52×10^−2^***	***1.45***	0.518	0.09	0.04
		***chr14.79312393***	***7.45×10^−6^***	***5.13***	0.514	−0.20	0.04	***2.37×10^−2^***	***1.63***	0.566	−0.10	0.04
Coat color, albino	0.967(0.04)*	***chr7.87642045***	***5.00×10^−106^***	***105.30***	0.432	−0.58	0.02	***1.59×10^−162^***	***161.80***	0.388	−0.61	0.02
Coat color, agouti	0.971(0.04)*	***chr2.154464466***	***9.43×10^−191^***	***190.03***	0.129	0.94	0.01	***5.7×10^−93^***	***92.24***	0.207	0.72	0.03
Locomotor test day 1, total distance traveled in 30min	0.968(0.24)*	chr19.21812298	3.98×10^−7^	6.40	0.461	−0.36	0.07	4.55×10^−1^	0.342	0.502	−0.05	0.06
Locomotor test day2, total distance traveled in 30min	0.988(0.19)*	chr8.17410225	5.65×10^−6^	5.248	0.171	0.42	0.09	8.34×10^−1^	0.079	0.202	0.02	0.08
			**F_3943_ GBS**					**F_34_ GBS replicate**				
Body weight	0.711(0.25)*	chr1.89192209	6.42×10^−6^	5.19	0.22	0.22	0.05	5.16×10^−2^	1.29	0.276	0.10	0.05
		***chr14.82586326***	***1.48×10^−6^***	***5.83***	0.658	−0.22	0.04	***3.08×10^−5^***	***4.51***	0.575	−0.19	0.05
Coat color, albino	0.967(0.04)*	***chr7.87255156***	***3.37×10^−166^***	***165.47***	0.389	−0.62	0.02	***7.80×10^−97^***	***96.11***	0.444	−0.57	0.02
Coat color, agouti	0.971(0.04)*	***chr2.155091628***	***1.78×10^−115^***	***114.75***	0.218	0.74	0.02	***1.51×10^−185^***	***184.82***	0.135	0.90	0.01
Locomotor test day 2, total distance traveled in 30min	0.988(0.19)*	chr15.67627183	3.33×10^−6^	5.478	0.461	0.30	0.06	2.07×10^−1^	0.683	0.522	−0.08	0.07

We found that using a broader definition of an association region rather than a single SNP did not improve replication between the F_34_ cohort and the F_39-43_ cohorts. Confidence intervals (*e.g.*, (Baud *et al.* 2013; Nicod *et al.* 2016)) and the LOD support interval (Conneally *et al.* 1985; Lander and Botstein 1989) have been used to define a QTL. LOD support interval is very sensitive to the density of the SNPs where sparse markers would produce misleadingly large support intervals. In contrast, the credible set interval is an estimate of the posterior probability for association at markers neighboring the discovery SNP, and thus defines the size of the association region. As a result, we extended the replication comparison from the discovery SNP position to the credible set interval. We found that in the replication cohort, the p-value at the discovery SNP and that at the top SNP within the credible set interval (defined by the discovery QTL) were generally similar (Table S10). The replication of the locus chr14.79312393 (discovered in the F_34_ cohort) in the F_39-43_ cohort was more successful using the discovery QTL region defined by the credible set interval; the p-value at the top SNP within the credible set interval was noticeably more significant (chr14.82586326; p-value = 1.48×10^−6^) than the p-value at the discovery SNP (chr14.79312393; p-value = 0.0237; Table S10). Our results suggest that for the most part, the discovery SNP accurately represented the association strength of the loci, presumably because of its strong linkage with the neighboring SNPs. In our case, defining a QTL region by the credible set interval did not increase the count of replicated sites between the two cohorts.

We then considered the more liberal “sign test”, a statistical method to test for consistent differences between pairs of observations, to determine whether the directions of the effect (beta) of the coat color, body weight and activity loci were in the same direction between the discovery and replication cohorts. Specifically, we compared whether the sign (direction) of the beta estimates are consistently above or below zero. We found that 11 of 12 comparisons passed this much less stringent test of replication. The one locus (at chr15.67627183) that did not pass the sign test was the locomotor locus “discovered” in F_39-43_ (Table 1).

In light of the failure to replicate the locomotor activity findings, we conducted a series of 2,500 simulations per trait to estimate the expected power of our replication cohorts. For each phenotype we used the kinship relatedness matrix and variance components estimated from the replication set. For the coat color traits, we found that we had 100% power to replicate the association at either genome-wide significant levels or the more liberal p < 0.05 threshold (Figure S6). For body weight and locomotor activity, power to replicate at a genome-wide significance threshold ranged from 20 to 85%, whereas power to replicate at the p < 0.05 threshold was between 80% and 100% (Figure S6). These power estimates were inconsistent with our empirical observations for the locomotor activity traits, none of which replicated at even the p < 0.05 threshold, where we should have had almost 100% power (Table 1; Figure S6). However, our power simulations did not account for Winner’s Curse (Zöllner and Pritchard 2007) or study-specific heterogeneity (Zou *et al.* 2019).

To determine whether these factors could explain the lower than expected rate of replication, we applied a statistical framework that jointly models Winner’s Curse and study-specific heterogeneity in two GWAS studies of the same phenotype (Zou *et al.* 2019). This framework proposes two random effects models. The first model (**WC**) only accounts for Winner’s Curse, while the second model accounts for both Winner’s Curse and study-specific heterogeneity due to confounding (**WC+C**). In this context, we define confounding as any biological or technical effect present in one study but not the other. We applied each of these models once using F_34_ as the discovery study and once using F_39-43_ as the discovery study. The models can be used to assess how well Winner’s Curse explains the observed levels of replication. For example, when F_34_ is used as the replication study for the albino coat color phenotype, the expected value of the replication summary statistics after accounting for winner’s curse is the same as the expected value after accounting for Winner’s Curse and confounding (Figure S7). While the 95% confidence intervals for the **WC+C** model are larger than the **WC** model, both models seem to explain the observed data well. However, when F_34_ is used as the discovery study for the locomotor activity on day 1 or body weight, the **WC+C** model explains the data better than the **WC** model.

In order to quantitatively assess how well each of these models explain the observed patterns of replication, we computed the predicted replication rates under each model (Methods) and compared these with the empirical replication rates. In this analysis, we defined the empirical replication rate to be the proportion of variants passing the genome-wide significance threshold in the discovery study that also pass the Bonferroni corrected threshold in the replication study. We used this definition of replication for this analysis instead of replication of lead SNPs to allow for a larger number of variants to be included in the model fitting process. For all phenotypes tested, the **WC** model predicts that all the variants passing the genome-wide significance threshold in the discovery study should pass the Bonferroni corrected threshold in the replication study, which is dramatically different from the observed replication of body weight and locomotor activity on day 1 and 2 phenotypes (Table 2). While the replication in the agouti coat color phenotype is not well predicted by the **WC+C** model, this may be due to the fact that the agouti phenotype is a dominant trait, while our model assumes additive allele effects. These results suggest that the sample sizes are sufficiently large that Winner’s Curse cannot account for the lack of replication. However, in these cases, the **WC+C** model has predicted replication rates that are much closer to the true (observed) values, indicating that the lack of replication in these phenotypes is more likely to be due to study-specific heterogeneity that is potentially caused by confounding.

**Table 2:** Predicted replication rates. We applied the replication analysis to phenotypes with at least two genome-wide significant variants in the discovery study. These phenotypes include body weight, albino coat color, agouti coat color, locomotor test day 1, and locomotor test day 2. We computed the true replication rate as the fraction of variants that were genome-wide significant in the discovery study that also passed the Bonferroni significance threshold in the replication study (“Empirical replication rate”). The model accounting for Winner’s Curse and confounding (“Predicted replication rate WC+C”) explains the true replication rate more accurately than the model accounting for only Winner’s Curse (“Predicted replication rate WC”)

Discovery set	Replication set	Phenotype	Empirical replication rate	Predicted replication rate (WC)	Predicted replication rate (WC+C)
**F_34_ GBS**	**F_39-43_ GBS**	Body weight	0.009	1.000	0.044
		Coat color, albino	1.000	1.000	0.997
		Coat color, agouti	0.932	1.000	0.577
		Locomotor test day 1	0.000	1.000	0.028
		Locomotor test day 2	0.000	1.000	0.140
**F_39-43_ GBS**	**F_34_ GBS**	Body weight	0.297	1.000	0.071
		Coat color, albino	0.911	1.000	0.932
		Coat color, agouti	0.815	1.000	0.925
		Locomotor test day 2	0.000	1.000	0.053

We evaluated whether or not the traits showed genetic correlations across the two cohorts. High genetic correlations would indicate a high degree of additive genetic effect that is shared between the two cohorts, and the low genetic correlations would indicate limited potential for replication. We used all autosomal SNPs to calculate genetic correlations between the F_34_ and F_39-43_ generations for body weight, coat color, and locomotor activity phenotypes (Table S11), using GCTA-GREML (Yang *et al.* 2011). Albino and agouti coat color, body weight and locomotor activity on days 1 and 2 were highly genetically correlated (r_G_ >0.7; Table S11). In contrast, locomotor activity on day 3 showed a significant but weaker genetic correlation (r_G_ = 0.577), perhaps reflecting variability in the quality of the methamphetamine injection, which were only given on day 3. Overall, these results suggest that genetic influences on these traits were largely similar in the two cohorts; however, the genetic correlations were less than 1, suggesting an additional barrier to replication that was not accounted for in our power simulations.

**Figure 3:**
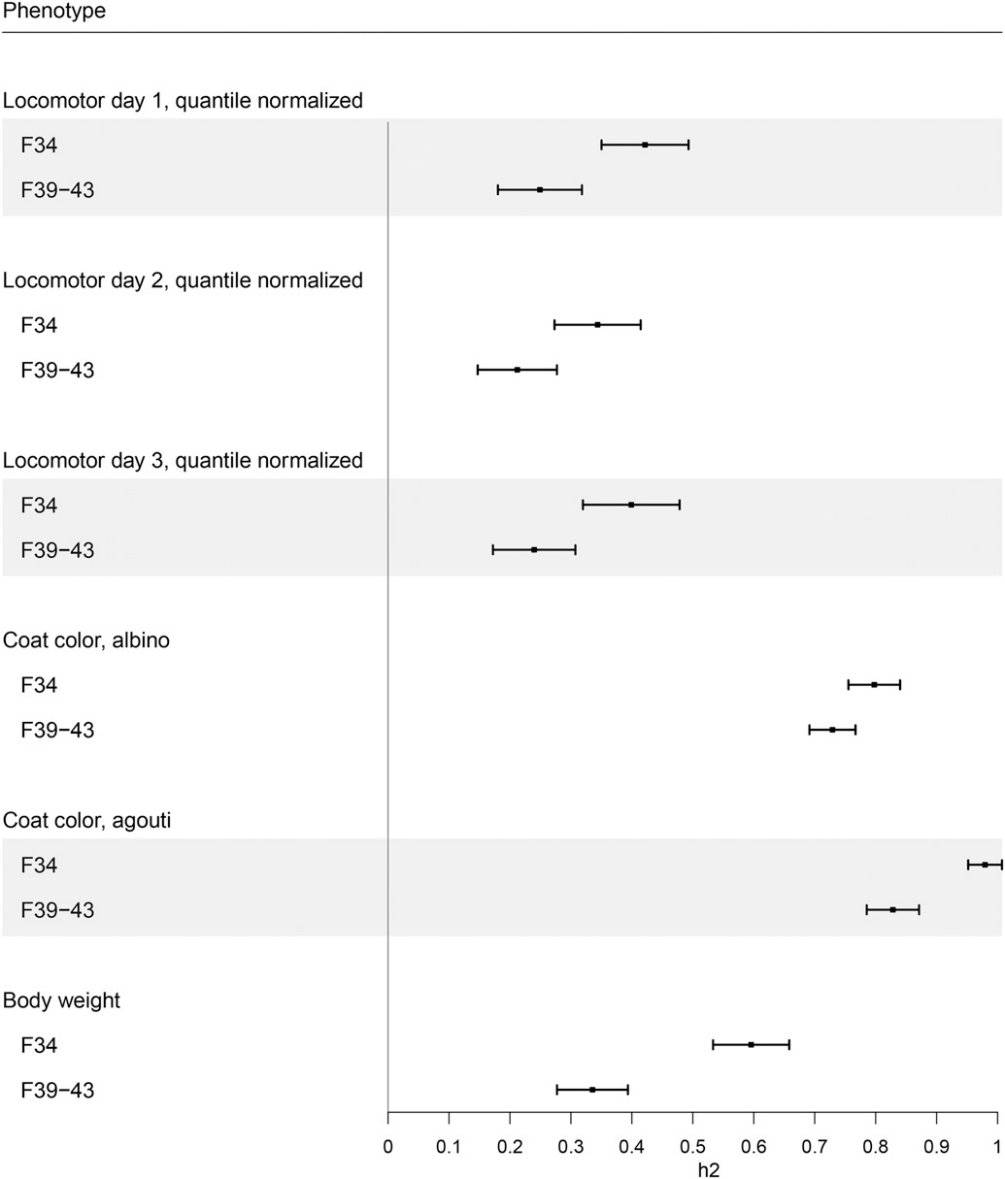
SNP-heritability estimates in F_34_ and F_39-43_ AILs. Square dots represent the SNP heritability estimated by the GCTA-GREML analysis (Yang *et al.* 2011). The whiskers flanking the square dots show the ± 1 × standard error of the heritability estimate. All heritability estimates are highly significant (p < 1.0×10^−05^; see Table S12).

We also calculated the SNP heritability for all traits using GCTA. SNP heritability was consistently lower in the F_39-43_ cohort compared to the F_34_ cohort, including the Mendelian traits of coat color. The ± 1 × standard error intervals of the F_34_ and F_39-43_ SNP heritability estimates for the coat color trait albino overlapped. This observation indicates that SNP heritability for albino in the two cohorts is comparable. In contrast, the ± 1 × standard error intervals of the F_34_ and F_39-43_ SNP heritability estimates for the coat color trait agouti did not overlap. We could not explain the differential SNP heritability for the binary trait agouti in the two cohorts. The lower SNP heritability in F_39-43_ for the rest of the quantitative traits could be a result of increased experimental variance (Figure 3; Table S12; (Falconer 1960; Lynch and Walsh 1996; Mhyre *et al.* 2005; Zöllner and Pritchard 2007; Visscher *et al.* 2008; Zaitlen and Kraft 2012)).

**Figure 4:**
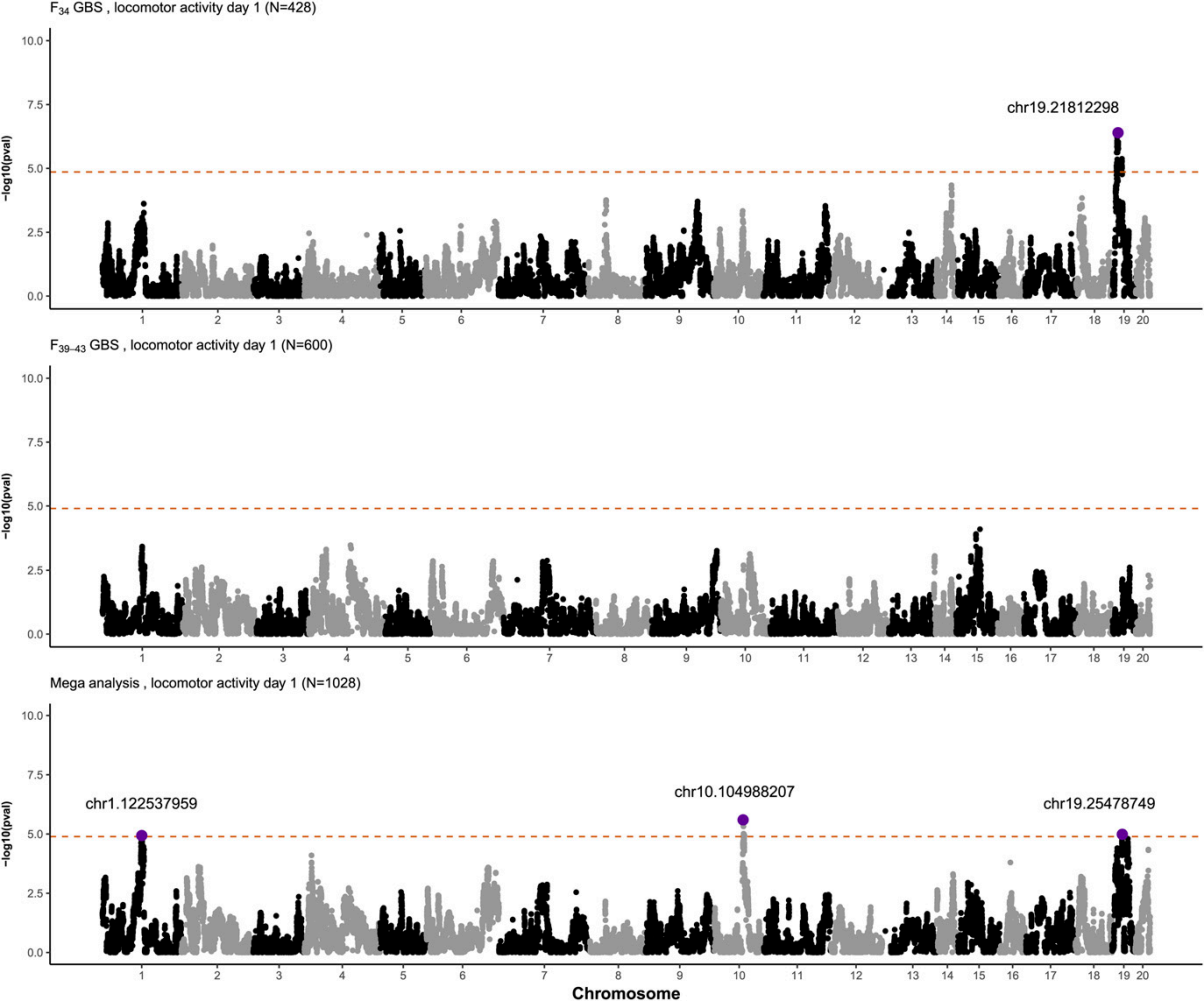
Manhattan plots comparing F_34_ GBS, F_39-43_ GBS, and mega-analysis on locomotor day 1 test using 57,170 shared SNPs in all AIL generations. We performed mega-analysis of F_34_ and F_39-43_ animals (n = 1,028) for body weight, coat color, and locomotor activity, the set of traits that were measured in the same way in both cohorts.

Due to the relatively high genetic correlations (Table S11), we suspected that a mega-analysis using the combined sample set would allow for the identification of additional loci; indeed, mega-analysis identified four novel genome-wide significant associations (Figure 4; Table S13). The significance level of five out of six loci identified by the mega-analysis was greater than that in either individual cohort. For instance, the p-values obtained by mega-analysis for chr14:82672838 (p-value = 7.93×10^−9^) for body weight were lower than the corresponding p-values for the same loci for F_34_ (chr14:79312393, p-value = 7.53×10^−6^) and F_39-43_ (chr14.82586326, p-value = 2.63×10^−6^; Table S13; Table 1).

## Discussion

We used F_34_ and F_39-43_ generations of a LG/J x SM/J AIL to perform GWAS, SNP heritability estimates, genetic correlations, replication and mega-analysis. We had previously performed several GWAS using a sparse marker set in the F_34_ cohort. In this study we used a denser set of SNPs, obtained using GBS, to reanalyze the F_34_ cohort. We found 109 significant loci, 36 of which had not been identified in our prior studies using the sparse marker set. We used a new, previously unpublished F_39-43_ cohort for GWAS and showed that genetic correlations were high for the subset of traits that were measured in both cohorts. Despite this, we found that many loci were not replicated between cohorts, even when we used a relatively liberal definition of replication (p < 0.05). The failure to replicate some of our findings was not predicted by our power simulations. Therefore, we performed an analysis to determine whether Winner’s Curse and study-specific heterogeneity could account for the lower than expected replication rate. Winner’s Curse alone could not explain the failure to replicate. However, modeling both Winner’s Curse and study-specific heterogeneity better explained the observed replication rate. Finally, mega-analysis of the two cohorts allowed us to discover four additional loci. Taken together, our results provide a set of refined regions of association for numerous physiological and behavioral traits in multiple generations of AILs. These loci could serve as benchmarks for future GWAS results in intercross mouse lines. More broadly, this study illustrates the difficulty of replication even when using a highly controlled model system.

Previous publications from our lab used a sparse set of array genotypes for GWAS of various behavioral and physiological traits in 688 F_34_ AILs (Cheng *et al.* 2010; Lionikas *et al.* 2010; Samocha *et al.* 2010; Parker *et al.* 2011, 2014; Carroll *et al.* 2017; Hernandez Cordero *et al.* 2018; Gonzales *et al.* 2018). In this study we obtained a much denser marker set for 428 of the initial 688 AIL mice using GBS. The denser genotypes allowed us to identify most of the loci obtained using the sparse set, as well as many additional loci. For instance, using the sparse markers we identified a significant locus on chromosome 8 for locomotor day 2 activity that contained only one gene: *Csmd1* (CUB and sushi multiple domains 1). Gonzales *et al.* (Gonzales *et al.* 2018) replicated this finding in F_50-56_ AILs and identified a *cis*-eQTL mapped to the same region. *Csmd1* mutant mice showed increased locomotor activity compared to wild-type and heterozygous mice, indicating that *Csmd1* is likely a causal gene for locomotor and related traits (Gonzales *et al.* 2018). We replicated this locus in the analysis of the F_34_ cohort that used the denser marker set (Figure S8). We also replicated a locus on chromosome 17 for distance traveled in the periphery in the open field test (Figure 4; (Parker *et al.* 2014)), three loci on chromosomes 4, 6, and 14 for body weight (Figure S8; (Parker *et al.* 2011)), one locus on chromosome 7 for mean corpuscular hemoglobin concentrations (MCHC, complete blood count; Figure S8; (Bartnikas *et al.* 2012)), and numerous loci on chromosome 4, 6, 7, 8, and 11 for muscle weights (Figure S8; (Lionikas *et al.* 2010)). We noticed that even using original sparse markers, some previously published loci were not replicated in the current GWAS. The most likely explanation is that we had only 428 of the 688 mice used in the previous publications.

QTL mapping studies have traditionally used a 1.0∼2.0 LOD support interval to approximate the size of the association region (see (Cervino *et al.* 2005; Logan *et al.* 2013)). The LOD support interval, proposed by Conneally *et al.* (Conneally *et al.* 1985) and Lander and Botstein (Lander and Botstein 1989), is a simple confidence interval method involving converting the p-value of the peak locus into a LOD score, subtracting “drop size” from the peak locus LOD score, and finding the two physical positions to the left and to the right of the peak locus location that correspond to the subtracted LOD score. Although Mangin *et al.* (Mangin *et al.* 1994) showed via simulation that the boundaries of LOD support intervals depend on effect size, others observed that a 1.0 ∼2.0 LOD support interval accurately captures ∼95% coverage of the true location of the loci when using a dense set of markers (Lander and Botstein 1989; Dupuis and Siegmund 1999; Manichaikul *et al.* 2006). In the present study, we considered using LOD support intervals but found that the sparse array SNPs produced misleadingly large support intervals. Various methods have been proposed for calculating confidence intervals in analogous situations (*e.g.*, (Baud *et al.* 2013; Nicod *et al.* 2016)). We performed credible set analysis and compared LocusZoom plots of the same locus region between array SNPs and the GBS SNPs (Figure S8; (Pruim *et al.* 2010)). For example, the benefit of the denser SNP coverage is easily observed in the locus on chromosome 7 (array lead SNP chr7:44560350; GBS lead SNP chr7:44630890) for the complete blood count trait “retic parameters cell hemoglobin concentration mean, repeat”; denser SNPs delineate the start and the end of an association block much more clearly. Thus, there are advantages of dense SNP sets that go beyond the ability to discover additional loci.

LD in the LG/J x SM/J AIL mice is more extensive than in the Diversity Outbred mice and Carworth Farms White mice (Parker *et al.* 2016). Some of the loci that we identified are relatively broad, making it difficult to infer which genes are responsible for the association. We focused on loci that contained five or fewer genes (Table S6). We highlight a few genes that are supported by the existing literature for their role in the corresponding traits. The lead SNP at chr1:77255381 is associated with tibia length in F_34_ AILs (Table S6; Figure S8). One gene at this locus, *EphA4*, codes for a receptor for membrane-bound ephrins. EPHA4 plays an important role in the activation of the tyrosine kinase JAK2 and the signal transducer and transcriptional activator STAT5B in muscle, promoting the synthesis of insulin-like growth factor 1 (IGF-1) (Lai *et al.* 2004; Hyun 2013; Sawada *et al.* 2017). Mice with mutated *EphA4* shows significant defect in body growth (Hyun 2013). Curiously, another gene at this locus, *Pax3*, has been shown as a transcription factor expressed in resident muscle progenitor cells and is essential for the formation of skeletal muscle in mice (Relaix *et al.* 2006). It is possible that both *EphA4* and *Pax3* are associated with the trait tibia length because they are both involved in organismal growth. Another region of interest is the locus at chr4:66866758, which is associated with body weight (Table S6; Table S13). The lead SNP is immediately upstream of *Tlr4*, Toll-like receptor 4, which recognizes Gram-negative bacteria by its cell wall component, lipopolysaccharide (Hoshino *et al.* 1999; Takeuchi *et al.* 1999). TLR4 responds to the high circulating level of fatty acids and induces inflammatory signaling, which leads to insulin resistance (Shi *et al.* 2006). Kim *et al.* showed TLR-4-deficient mice were protected from the increase in proinflammatory cytokine level and gained less weight than wild-type mice when fed on high fat diet (Kim *et al.* 2012). The association between *Tlr4* and body weight in the AILs corroborates these findings.

We considered both the F_34_ and the F_39-43_ as both “discovery” and “replication” cohorts. Significant loci for coat color, which are monogenic and served as positive controls, were replicated, between the two cohorts, as expected. One locus for body weight was replicated (p < 0.05) between F_34_ and F_39-43._ However, the loci for locomotor activity were not replicated. Power analyses predicted a much higher rate of replication, which led us to conduct additional analyses to better understand the lower than expected rate of replication.

First, we used a newly introduced method to determine whether Winner’s Curse (Zöllner and Pritchard 2007)) which has also been termed the Beavis Effect (Beavis *et al.* 1991, 1994; Xu 2003; King and Long 2017; Keele *et al.* 2019; Paterson 2019) could account for the lower than expected rate of replication. Beavis’ original report described a lack of replication of QTL for agronomic traits between small populations of maize (Beavis *et al.* 1991). Using progeny sizes ranging from 100 to 1000, Beavis simulated interval mapping to evaluate the accuracy of the estimates of phenotypic variance explained at the statistically significant QTL (Beavis *et al.* 1994; Xu 2003; Paterson 2019). Simulations showed that progeny sizes greatly influenced the estimates of phenotypic variance explained; smaller progeny sizes (n = 100) generated highly overestimated estimates of phenotypic variances, whereas larger progeny sizes (n = 1000) generated estimates of phenotypic variances similar to the actual value (Xu 2003; Paterson 2019). King and Long (King and Long 2017) further examined the Beavis Effect in the next-generation mapping populations in *Drosophila melanogaster*. The authors found that sample size was the major determinant for the overestimation of phenotypic variance explained at the significant QTL in both the GWAS-based Drosophila Genetic Reference Panel (DGRP) and the multi-parental Drosophila Synthetic Population Resource (DSPR). When sample size remained constant and the true phenotypic variance explained at the significant QTL was small, the estimation bias was more pronounced. In contrast, estimates for the phenotypic variance explained at all simulated QTL, significant or not, were generally centered at the true values. In an analogous study of power and replication in Collaborative Cross mice, Keele *et al.* (Keele *et al.* 2019) found that the Beavis Effect was most striking when the number of strains and true effect size of the QTL were small. This estimation bias indicates that mapping statistically significant QTL across experiments, populations, and panels can be problematic (Macdonald and Long 2004; Gruber *et al.* 2007; Najarro *et al.* 2015). The analyses we performed indicated that Winner’s Curse alone could not explain the lack of replication, but a model that also included study-specific heterogeneity could.

Our analysis does cannot explain the source of the study-specific heterogeneity. Possible sources of confounding could include maternal effects, which could differentiate the F_34_ cohort and the F_39-43_ cohort because F_33_ animals were transported to the University of Chicago from Washington University in St. Louis. In contrast, the breeder animals of the F_39-43_ cohort have already acclimated to the environment for multiple generations. Another possible source of confounding is that the phenotyping of the F_39-43_ occurred over five generations (more than a year) during which time numerous environmental factors may have changed (*e.g.*, several technicians performed the data collection). Such factors are known to be an important potential source of confounding; (Falconer 1960; Lynch and Walsh 1996; Crabbe *et al.* 1999; Mhyre *et al.* 2005; Visscher *et al.* 2008; Zaitlen and Kraft 2012; Sorge *et al.* 2014). Our analyses did not correct for the fact that six phenotypes were examined, thus somewhat increasing the chances that at least one of our significant associations could have been a false positive that would not be expected to replicate.

Interestingly, we found that the genetic correlations between the discovery and replication samples were relatively high for all traits; however, some traits replicated well and others replicated poorly. Our subsequent analysis showed that study-specific heterogeneity was low for the coat color traits, but higher for the body weight and locomotor traits. This makes an important point, namely that a strong genetic correlation can exists in the presence or absence of study-specific heterogeneity. Finally, it was notable that replication (at p < 0.05) was relatively successful for body weight, despite the significant evidence of study-specific heterogeneity and low predicted replication (Table 2). Power analyses predicted that the body weight loci should replicate at the genome-wide significance threshold, whereas we only observed replication when at the less stringent p < 0.05 level (Table 1). The lack of replication at the genome-wide significance threshold for the body weight phenotype was likely due to study-specific heterogeneity due to confounding that was not accounted for in the power analyses. In Table 2, “predicted replication” refers to replication using a Bonferroni significance threshold that accounts for the number of significant SNPs in the discovery study. The low predicted replication rate under the **WC+C** model for the body weight phenotype is consistent with the low replication (genome-wide) reported in Table 1. Thus, both body weight and locomotor traits were strongly impacted by study specific confounding; however, nominal replication was still possible for body weight but not for the locomotor traits.

Finally, we performed a mega-analysis using F_34_ and F_39-43_ AIL mice. The combined dataset allowed us to identify four novel genome-wide significant associations that were not detected in either the F_34_ or the F_39-43_ cohorts presumably because of the increased sample size in the mega-analysis (Visscher *et al.* 2017). As is true for all GWAS, the loci identified in the mega-analysis could be false positives.

In addition to performing many GWAS and related analyses that led to the identification of dozens of novel loci for locomotor activity, open field test, fear conditioning, light dark test for anxiety, complete blood count, iron content in liver and spleen, and muscle weight, our study also tested our expectations about replication of GWAS findings. We did not obtain the expected rate of replication. We used a method that can distinguish between Winner’s Curse and study-specific heterogeneity to show that the lower than expected rate of replication was due to study-specific heterogeneity. This indicates that study-specific heterogeneity can have a major impact of replication even when in a model system when a genetically identical population is tested under conditions that are designed to be as similar as possible.
